# Electronic cigarettes for smoking cessation: An overview of systematic reviews and evidence and gap map

**DOI:** 10.1111/add.70388

**Published:** 2026-03-26

**Authors:** Angela Difeng Wu, Monserrat Conde, Ailsa R. Butler, Ethan Knight, Nicola Lindson, Jonathan Livingstone‐Banks, Peter Hajek, Hayden McRobbie, Rachna Begh, Annika Theodoulou, Caitlin Notley, Tari Turner, Eliza Zhitnik, Jamie Hartmann‐Boyce

**Affiliations:** ^1^ Nuffield Department of Primary Care Health Sciences University of Oxford Oxford UK; ^2^ Wolfson Institute of Population Health Queen Mary University of London London UK; ^3^ National Drug and Alcohol Research Centre University of New South Wales Sydney Australia; ^4^ Norwich Medical School University of East Anglia Norwich UK; ^5^ Cochrane Australia School of Public Health & Preventive Medicine Melbourne Australia; ^6^ Department of Health Promotion and Policy University of Massachusetts Amherst MA USA

**Keywords:** electronic cigarettes, evidence and gap map, evidence synthesis, overview of review, smoking, smoking cessation, tobacco, vapes

## Abstract

**Background and aims:**

Electronic cigarettes (EC) are considered a smoking cessation tool in some countries, such as the United Kingdom, but uncertainty remains internationally over whether their benefits outweigh potential harms when used for this purpose. This overview (1) synthesised existing evidence from systematic reviews (SR) on the effectiveness and safety of ECs to explore and address these uncertainties and disagreements and (2) mapped primary intervention studies to identify priorities for further research.

**Methods:**

Overview of SRs published from 1 January 2015 and meeting the inclusion criteria of the Cochrane review of EC for smoking cessation. We searched seven databases to April 2024. We followed Cochrane screening and data extraction methods. We adapted Campbell Collaboration and 3ie methods for the Evidence and Gap Map (EGM). We assessed review quality using AMSTAR‐2.

**Results:**

We included 14 reviews of intervention studies (7 high quality; 7 low quality), with search dates from 2014 to 2023, in adult populations including the general population, people at risk of lung cancer, with comorbid health conditions and pregnant people. Eighteen studies were included across multiple reviews, some of which included multiple meta‐analyses. Across 21 meta‐analytic comparisons of nicotine EC versus other interventions, all reported point estimates favouring nicotine EC for smoking cessation, with relative risks/odds ratios typically in the range 1.17–1.67 versus nicotine replacement therapy and 1.46–2.09 versus non‐nicotine EC, with higher‐quality reviews giving more consistent estimates. Of 13 reviews that meta‐analysed serious adverse events (SAEs), two reported point estimates suggesting increased SAEs with nicotine EC; other estimates included the possibility of no difference. For adverse events, pooled estimates generally indicated little or no difference between groups. Our EGM mapped 90 primary, complete studies and identified absolute gaps in evidence comparing the effects of nicotine EC to cytisine, bupropion and nicotine pouches. Most studies used collected data from high‐income countries.

**Conclusion:**

Meta‐analyses of electronic cigarettes (EC) for smoking cessation report point estimates favouring higher ≥6‐month smoking cessation rates with nicotine EC compared with nicotine replacement therapy, non‐nicotine EC/placebo, behavioural or no support and mixed support. Evidence on serious adverse events (SAEs) remains inconclusive. Evidence gaps were identified in SAE data and in studies from low‐ and middle‐income countries.

## INTRODUCTION

Electronic cigarettes (ECs) are handheld electronic devices that produce an aerosol by heating an e‐liquid. The e‐liquid, usually comprising propylene glycol and glycerol, with or without nicotine and flavours, is stored in disposable or refillable cartridges or a reservoir or ‘pod’. People who smoke, healthcare providers and regulators want to know if ECs can help people quit smoking, and if they are safe to use for this purpose. Smoking remains the leading preventable cause of cancer, death and health inequalities in the UK and worldwide [1, 2, 3]. ECs are considerably less harmful than tobacco cigarettes, and in the UK are endorsed by the National Institute for Health and Care Excellence, the Office for Health Improvement and Disparities and The Royal College of Physicians as smoking cessation aids [4, 5]. Despite endorsement by some bodies in England, uncertainties and perceived controversies about their role in reducing smoking hamper international policy and clinical/personal decision‐making.

An overview of systematic reviews, or systematic review of reviews, brings together and summarises existing evidence from multiple systematic reviews and makes the findings more accessible. Overviews also play a valuable role where conclusions from systematic reviews on the same topic conflict; an overview can bring together the findings and appraise and analyse the evidence in a systematic way [6]. Evidence and gap maps (EGMs) are interactive web‐based tools that offer a visual representation of clusters of, and gaps in, evidence in a specific field of research. They can supply stakeholders with information to support decision‐making in both policy and research prioritisation [7, 8, 9].

We set out to conduct an overview of systematic reviews and an EGM of reviews and primary studies investigating ECs for smoking cessation, to clarify areas of consensus, areas of disagreement and gaps in the available literature.

## METHODS

This overview of reviews and EGM builds on the work of the Cochrane living systematic review (LSR) of ECs for smoking cessation [10]. We reported our work according to guidelines for the Preferred Reporting Items for Systematic Reviews and Meta‐Analyses (PRISMA) [11], as per journal guidance. We pre‐registered our protocol on the Open Science Framework: https://osf.io/unpb3.

### Inclusion criteria

Eligibility criteria for both the overview and the EGM followed the population, intervention, comparator, outcomes and study‐design framework used in the Cochrane LSR [10] (explicitly stated in Table 1).

**TABLE 1:** Inclusion criteria.

Domain	Overview	EGM
Population/participants	People defined as currently using combustible cigarettes at study enrolment. Participants could be motivated or unmotivated to quit. We included studies that recruit pregnant people	
Intervention	Any type of ECs or intervention intended to promote EC use for smoking cessation, including studies that did not measure smoking cessation but provided ECs with the instruction that they be used as a complete substitute for cigarette use. ECs may or may not contain nicotine	
Comparators	Alternative smoking cessation aids, including NRT or no intervention, ECs without nicotine, other variations on ECs and minimal support/no support	
Outcomes	Smoking cessation at 6 months or more. Any of the following outcomes at 1 week or more: adverse events; serious adverse events; heart rate; blood pressure; carbon monoxide; lung function test; oxygen saturation; known toxins/carcinogens, as measured through blood or urine	
Study types	Completed systematic reviews of intervention studies (excluding reviews including observational data), meeting PRISMA definition for systematic reviews and published from 1 January 2015 onwards [12]. Studies were considered systematic reviews if they report: (i) research question; (ii) reproducible search strategy, including search databases; (iii) screening methods and inclusion and exclusion criteria; (iv) quality appraisal methods; and (v) reporting of data analysis [13].	As per overview, plus randomised controlled trials (including crossover trials), and uncontrolled intervention studies where all participants are given an EC intervention, as per Lindson 2024 [10]

Abbreviations: EC = electronic cigarette; NRT = nicotine replacement therapy; PRISMA = Preferred Reporting Items for Systematic Reviews and Meta‐Analyses.

### Searches and study selection

An information specialist designed and conducted systematic searches in the following databases from 1 January 2004 (just prior to ECs appearing on the global market) to 3 April 2024:

- Cochrane Database of Systematic Reviews;
- Cochrane Tobacco Addiction Group Specialised Register (not searched beyond 1 February 2023, when the database stopped being maintained);
- Cochrane Central Register of Controlled Trials (CENTRAL, via CRS‐Web);
- MEDLINE (via OvidSP);
- Embase (via OvidSP);
- PsycINFO (via OvidSP);
- ClinicalTrials.gov (via CENTRAL);
- WHO International Clinical Trials Registry Platform (ICTRP, www.who.int/ictrp/en/, via CENTRAL).

The search strategy was based on our companion Cochrane review [14] and is available in Appendix S1.

Results were imported into Covidence and screened independently by two reviewers, with discrepancies resolved through discussion or by a third reviewer.

### Critical appraisal of systematic reviews

The quality of each included systematic review was evaluated using the key domains of the AMSTAR‐2 (A MeaSurement Tool to Assess systematic Reviews, revised) checklist [15]. Reviews were categorised as either ‘higher’ or ‘lower’ quality depending on whether they achieved a rating of ‘yes’ or ‘partial yes’ in at least six of the seven critical AMSTAR‐2 domains, following methodology described elsewhere [16]. Reviews judged as ‘yes’ or ‘partial yes’ for six or seven critical domains were considered higher quality, while those judged as ‘no’ for at least two critical domains were considered lower quality. These critical domains included the existence of and adherence to a protocol, the use of a comprehensive search strategy, the provision of a justified list of excluded studies, the use of a satisfactory risk‐of‐bias assessment technique, the use of appropriate meta‐analytical methods, the consideration of risk of bias in discussing the results of the review and the adequate investigation of publication bias, where appropriate. Lower‐quality reviews were included in the overview, but their limitations were considered when interpreting the findings.

### Overview of reviews: data extraction, synthesis and analysis

Data extraction and critical appraisal were performed by a primary reviewer using a pre‐defined and tested data extraction form, with verification by a secondary reviewer (template data extraction sheet available in Appendix S2). Discrepancies were resolved through discussion or by consulting a third reviewer.

We narratively synthesised information on the included studies and the conclusions of each review. We noted whether studies were included in multiple reviews to account for potential overlap, and for reviews that included more than 60 primary studies we only checked the overlap for primary studies relating to ECs. For quantitative reviews, we reported pooled estimates, 95% confidence intervals (95% CIs) and *I* ^2^ values as indicators of statistical inconsistency, along with any critical appraisal findings or authors’ certainty judgements (e.g. Grading of Recommendations Assessment, Development and Evaluation, GRADE). Findings were grouped by outcome and population. Where reviews contributed meta‐analysis data to an outcome, this was charted in effect/association direction plots, following Synthesis Without Meta‐analysis (SWiM) guidance [17]. Effect‐direction plots were used for visual summary only and not for quantitative inference.

### EGM framework and data coding

We adapted EGM methods from the Campbell collaboration guidance [9] and 3ie (International Initiative for Impact evaluation) [8]. We designed our draft framework according to the linked living Cochrane review [14]. The overall research project was also informed by patient and public involvement and input. We developed a coding data set on EPPI‐Reviewer (Evidence for Policy and Practice Information Centre, London, UK) and replicated it on an MS Excel (Microsoft, Redmond, WA, USA) spreadsheet. Coding of primary studies and systematic reviews was conducted in duplicate using MS Excel spreadsheets and checked by a third reviewer. The finalised coding data set was then inputted to EPPI‐Reviewer by an experienced reviewer. We produced a coding report that was then imported to EPPI‐Mapper. For the coding categories, see Appendix S3; all definitions of the EGM categories and filters can be found in the EGM glossary (Appendix S3).

We generated our interactive EGM with the EPPI‐Mapper software. The EGM matrix shows comparators as rows and outcomes as columns. The ‘bubbles’ map view shows by default. Included studies related to that combination of comparator and outcome appear as circles or gaps (empty cells). The size of the ‘bubble’ is proportional to the number of studies identified. Empty cells are interpreted as absolute gaps in evidence, whereas few studies in a cell represent a relative gap. By applying the filters, users can customise their searches of the evidence identified based on the characteristics of interest (e.g. country). Clicking a ‘bubble’ opens a tab displaying a list of related references. Users can view the summary details and URL for an article by selecting a reference. We analysed the included studies descriptively across study design and distribution by comparator types and outcomes.

## RESULTS: OVERVIEW OF REVIEWS

### Characteristics of included reviews

We included 14 reviews, including 109 primary studies (including 21 studies not related to ECs). See Appendix S1 for the PRISMA flow diagram and Table 2 for characteristics of the included reviews. Eighteen studies were included in multiple reviews (Table 3).

**TABLE 2:** Characteristics of included reviews.

Review	Number of included studies	Number of included participants	Latest search date	Country(ies)	Industry funding	Overall AMSTAR‐2 rating	Comparators reported	Outcomes reported
Chan 2021 [18]	16^a^	11 754	Apr 2020	New Zealand, Italy, USA, UK, Republic of Korea, Canada, Hong Kong, Iran, the Netherlands, Denmark, Germany and Finland	No declared industry funding	Lower Quality	Nicotine ECs vs NRT, nicotine ECs vs nicotine‐free control (placebo EC or non‐pharma support)	Smoking cessation at ≥6 months
Hanewinkel 2022 [19]	4	1598	Jul 2021	New Zealand, Australia, UK and USA	No declared industry funding	Lower Quality	Nicotine ECs vs NRT	Smoking cessation at ≥6 months
Huang 2023 [20]	8	988	Jan 2022	England, Canada, Italy, America and Australia	No declared industry funding	Lower Quality	Nicotine ECs vs behavioural support only/no support	Smoking cessation at ≥6 months, AEs at 24 weeks (6 months)
Ibrahim 2021 [21]	12	9863	Dec 2019	Unable to access	No declared industry funding	Higher Quality	Nicotine ECs vs NRT	Smoking cessation at ≥6 months, AEs at 24 weeks (6 months), SAEs at 24 weeks (6 months)
Khoudigian 2016 [22]	5	840	May 2014	New Zealand, UK and Italy	No declared industry funding	Lower Quality	Nicotine ECs vs non‐nicotine ECs	Smoking cessation at ≥6 months, AEs at 24 weeks (6 months)
Levett 2023 [23]	5	3253	Jan 2022	Canada, Italy, New Zealand and UK	No declared industry funding	Higher Quality	Nicotine ECs vs non‐EC intervention, nicotine ECs vs non‐nicotine ECs, non‐nicotine ECs vs non‐EC intervention	Smoking cessation at ≥6 months, SAEs at 24 weeks (6 months)
Li 2022 [24]	5	1748	Oct 2021	USA, Republic of Korea, New Zealand, UK and Australia	No declared industry funding	Higher Quality	Nicotine ECs vs NRT	Smoking cessation at ≥6 months, AEs at 24 weeks (6 months), SAEs at 24 weeks (6 months)
Lindson 2023 [25]	319^a^	157 179	Apr 2022	Europe, USA, Canada, Australasia, Bangladesh, China, Egypt, Iceland, India, Iran, Israel, Japan, Kyrgyzstan, Pakistan, Republic of Korea, South America, Syria, Taiwan, Thailand and Turkey	No declared industry funding	Higher Quality	Nicotine ECs vs placebo, nicotine ECs vs NRT, nicotine ECs vs non‐nicotine ECs, nicotine ECs vs behavioural support/no support, nicotine ECs + NRT vs NRT, nicotine ECs + NRT vs non‐nicotine ECs + NRT	Smoking cessation at six months or longer, SAEs at 1 week
Lindson 2024 [10]	88	27 235	Jul 2023	USA, UK, Italy, Australia, Greece, New Zealand, Switzerland, Canada, Belgium, Ireland, the Netherlands, Poland, Republic of Korea, South Africa and Turkey	No declared industry funding	Higher Quality	Nicotine ECs vs NRT, nicotine ECs vs non‐nicotine ECs, nicotine ECs vs behavioural support/no support, nicotine ECs vs heated tobacco, nicotine ECs vs varenicline, nicotine ECs + NRT vs NRT, nicotine ECs + NRT vs non‐nicotine ECs + NRT	Smoking cessation at ≥6 months, AEs at 1, 8, 12 and 24 weeks (6 months), blood oxygen saturation at ≥1 week, carbon monoxide (ppm) at ≥1 week, carbon monoxide (ppm) at ≥1 week, heart rate (bpm) at ≥1 week, lung function (e.g. FEV) at ≥1 week or longer, SAEs at 1, 4, 8 and 24 weeks (6 months), study product use at ≥1 week and at ≥6 months, systolic blood pressure at ≥1 week, systolic blood pressure at ≥1 week, toxicants (e.g. 3‐HPMA, 2‐HPMA, AAMA, HMPMA, NNAL) at ≥1 week
Patnode 2021 [26]	9	35 665	Sep 2020	New Zealand, Italy, UK, Korea and USA	No declared industry funding	Lower Quality	Nicotine ECs vs non‐EC interventions	Smoking cessation at ≥6 months
Pound 2021 [27]	6	3942	Jun 2020	Australia, UK, Republic of Korea and USA	No declared industry funding	Higher Quality	Nicotine ECs vs NRT	Smoking cessation at ≥6 months, AEs at 24 weeks (6 months)
Quigley 2021 [28]	10	1996	May 2021	Italy, UK, USA, South Korea, Canada and New Zealand	No declared industry funding	Lower Quality	Nicotine ECs vs NRT, nicotine ECs vs placebo ECs, nicotine ECs vs non‐pharma support	Smoking cessation at ≥6 months, AEs at 24 weeks (6 months)
Thomas 2021 [29]	363^a^	4853	Feb 2019	USA, UK and multi‐country	No declared industry funding	Higher Quality	Nicotine ECs (high/low dose) vs placebo	Smoking cessation at ≥6 months
Vanderkam 2022 [30]	7	201 045	Mar 2022	Italy, New Zealand, Canada, UK and Korea	No information	Lower Quality	Nicotine ECs vs NRT, nicotine ECs vs non‐nicotine ECs	Smoking cessation at ≥6 months

Abbreviations: AE = adverse event; EC = electronic cigarette; FEV = forced expiratory volume; NRT = nicotine replacement therapy; SAE = serious adverse event.

^a^ Includes number of non‐EC related primary studies.

**TABLE 3:** Overlap of studies across included reviews.

Study/Systematic Review	Lindson	Chan	Hanewinkel	Huang	Ibrahim	Khoudigian	Levett	Li	Patnode	Pound	Quigley	Thomas	Vanderkam	Total
	2024	2021	2022	2023	2021	2016	2023	2022	2021	2021	2021	2021	2022	
Adriaens 2014														2
Baldassarri 2018														3
Bullen 2013														12
Caponnetto 2013a														8
Carpenter 2017														4
Cobb 2021														2
Eisenberg 2020														4
Eisenhofer 2015														2
Hajek 2019														11
Halpern 2018														5
Hatsukami 2020														3
Holliday 2019														2
Lee 2018														7
Lee 2019														8
Lucchiari 2022														3
Masiero 2019														5
Tseng 2016														3
Walker 2020														4
Total	18	7	3	1	11	2	5	4	8	6	10	6	7	

### Quality of included reviews

Seven reviews were higher quality and seven were lower quality (Table 4). The most frequently unmet AMSTAR‐2 domains were not listing excluded studies with reasons (10 reviews), not considering risk of bias when interpreting findings (5) and not investigating publication bias where quantitative synthesis was performed (7). Two reviews lacked a pre‐registered protocol and three used inappropriate meta‐analytic methods.

**TABLE 4:** AMSTAR‐2 (A MeaSurement Tool to Assess systematic Reviews, revised) summary.

Study	Chan	Hanewinkel	Huang	Ibrahim	Khoudigian	Levett	Li	Lindson	Lindson	Patnode	Pound	Quigley	Thomas	Vanderkam
	2021	2022	2023	2021	2016	2023	2022	2023	2024	2021	2021	2021	2021	2022
Pre‐registered methods & justifications	Yes	Yes	Yes	Partial Yes	No	Yes	Yes	Yes	Yes	Yes	Yes	No	Yes	No
Comprehensive literature search	Yes	Partial Yes	Partial Yes	Partial Yes	Partial Yes	Partial Yes	Partial Yes	Yes	Yes	Yes	Yes	Partial Yes	Yes	Partial Yes
Excluded studies listed & justified	No	No	No	Yes	No	No	No	Yes	Yes	No	No	No	Yes	No
Risk of bias assessed	Yes	Yes	Yes	Yes	Yes	Yes	Yes	Yes	Yes	Partial Yes	Yes	Yes	Yes	Yes
Appropriate meta‐analysis methods	Yes	Yes	No	Yes	Yes	Yes	Yes	Yes	Yes	No	Yes	Yes	Yes	No
Risk of bias considered in findings	No	Yes	No	Yes	No	Yes	Yes	Yes	Yes	No	Yes	Yes	Yes	No
Publication bias investigated	Yes	No	No	Yes	No	Yes	Yes	Yes	Yes	No	Yes	No	No	No
**AMSTAR‐2 rating**	Critically low	Critically low	Critically low	High	Critically low	Low	Low	High	High	Critically low	Low	Critically low	Low	Critically low
**Overall grade**	Lower Quality	Lower Quality	Lower Quality	Higher Quality	Lower Quality	Higher Quality	Higher Quality	Higher Quality	Higher Quality	Lower Quality	Higher Quality	Lower Quality	Higher Quality	Lower Quality

### Smoking cessation at 6 months or more

Table 5 summarises the findings from meta‐analyses and whether 95% CIs included the possibility of no difference. Across 21 comparisons of nicotine ECs with other interventions, all reviews reported point estimates favouring nicotine ECs, though the effect size and precision varied.

**TABLE 5:** Effect direction plot, smoking cessation.

Comparator	Review	Analysis type	Smoking cessation	Effect size (estimate and range)	AMSTAR quality
Nicotine ECs vs NRT	Chan (2021)	NMA	▲	RR = 1.49 (1.09–2.04)	Lower
	Hanewinkel (2022)	PW	▲	RR = 1.58 (1.20–2.08)	Higher
	Ibrahim (2021)	PW	△	RR = 1.35 (0.95–1.90)	Higher
	Li (2022)	PW	▲	RR = 1.67 (1.21–2.28)	Lower
	Lindson (2024)	PW	▲	RR = 1.59 (1.30–1.93)	Higher
	Pound (2021)	PW	△	RR = 1.42 (0.97–2.09)	Lower
	Quigley (2021)	NMA	◁▷	RR = 1.17 (0.65–1.86)	Lower
	Vanderkam (2022)	PW	▲	RR = 1.49 (1.14–1.95)	Lower
Nicotine ECs vs non‐nicotine ECs/placebo	Khoudigian (2016)	PW	△	RR = 2.02 (0.97–4.22)	Lower
	Levett (2023)	PW	▲	RR = 1.56 (1.13–2.15)	Higher
	Lindson (2024)	PW	▲	RR = 1.46 (1.09–1.96)	Higher
	Vanderkam (2022)	PW	▲	RR = 1.66 (1.01–2.73)	Lower
	Chan (2021)	NMA	▲	RR = 2.09 (1.46–2.99)	Lower
	Lindson (2023)	CNMA	▲	OR = 2.37 (1.73–3.24)	Higher
High‐dose nicotine ECs vs control	Thomas (2021)	NMA	▲	OR = 3.22 (1.63–6.36)	Higher
Low‐dose nicotine ECs vs control	Thomas (2021)	NMA	△	OR = 3.22 (0.97–12.55)	Higher
Nicotine ECs vs behavioural or no support	Huang (2023)	PW	▲	RR = 1.51 (1.03–2.21)	Lower
	Lindson (2024)	PW	▲	RR = 1.88 (1.56–2.25)	Higher
Nicotine ECs vs mixed interventions	Levett (2023)	PW	▲	RR = 1.77 (1.29–2.44)	Higher
Nicotine ECs + NRT vs non‐nicotine ECs + NRT	Lindson (2024)	PW	▲	RR = 1.77 (1.07–2.94)	Higher
Nicotine ECs + NRT vs NRT	Lindson (2024)	PW	▲	RR = 3.53 (1.93–6.44)	Higher
Non‐nicotine ECs vs NRT	Lindson (2024)	PW	◁▷	RR = 0.99 (0.64–1.54)	Higher
	Quigley (2021)	NMA	▽	RR = 0.65 (0.27–1.30)	Lower
Non‐nicotine ECs vs mixed interventions	Levett (2023)	PW	△	RR = 1.37 (0.66–2.85)	Higher
Non‐nicotine ECs vs behavioural support only/no support	Lindson (2024)	PW	△	RR = 1.63 (0.81–3.25)	Higher

*Note*: A varenicline comparator was not included in the table as there were no meta‐analyses for that comparison, the one comparison noted included only one study. ▲ = greater quit rates in EC arm, 95% CI does not include possibility of no difference; ▼ = fewer quit rates in EC arm, 95% CI does not include possibility of no difference; ◁▷ = no change/mixed effects/conflicting findings; △ = greater quit rates in EC arm, but 95% CI includes possibility of no difference; ▽ = fewer quit rates in EC arm, but 95% CI includes possibility of no difference; AMSTAR = A MeaSurement Tool to Assess systematic Reviews; CNMA = component network meta‐analysis; EC = electronic cigarette; NMA = network meta‐analysis; NRT = nicotine replacement therapy; PW = pairwise comparison.

#### Nicotine ECs versus nicotine replacement therapy (NRT)

Eight systematic reviews compared nicotine‐containing ECs with NRT.

Among the higher‐quality reviews, Hanewinkel [19] (4 studies, *n* = 1598) reported a relative risk (RR) of 1.58 (95% CI = 1.20–2.08, *I* ^2^ = 8%), and Lindson [10] (7 studies, *n* = 2544) found a similar estimate of 1.59 (95% CI = 1.30–1.93, *I* ^2^ = 0%). Ibrahim [21] (7 studies, *n* = 5435) reported an RR of 1.35 (95% CI = 0.95–1.90, *I* ^2^ = 13%), with the 95% CI encompassing modest benefit and no difference.

Among the lower‐quality reviews, Li [24] (2 studies, *n* = 1468) reported an RR of 1.67 (95% CI = 1.21–2.28, *I* ^2^ = 6%), Vanderkam [30] (3 studies, *n* = 1605) found an RR of 1.49 (95% CI = 1.14–1.95, *I* ^2^ = 70%), indicating substantial inconsistency across studies, Chan [18] (network meta‐analysis; 4 studies, *n* not reported) estimated an RR of 1.49 (95% CI = 1.09–2.04, *I* ^2^ = 42%), Pound [27] (5 studies, *n* = 1800) reported an RR of 1.42 (95% CI = 0.97–2.09, *I* ^2^ = 50%) and Quigley [28] (network meta‐analysis; 8 studies, *n* not reported) found an RR of 1.17 (95% CI = 0.65–1.86).

Across these reviews, estimates ranged from 1.17 to 1.67, favouring nicotine‐containing ECs over NRT. However, the 95% CIs in several reviews often included values consistent with small benefits and no clear difference, reflecting uncertainty in the precise size of the effect. The direction of effect was similar across higher‐ and lower‐quality reviews.

Lindson [10] and Hanewinkel [19] graded their evidence as high and moderate certainty, respectively. In contrast, Pound [27], Quigley [28] and Li [24] judged theirs to be low or very low certainty, most often owing to inconsistency (as indicated by *I* ^2^), reliance on self‐reported cessation outcomes or small sample sizes.

#### Nicotine ECs versus non‐nicotine ECs/placebo

Seven systematic reviews evaluated the effectiveness of nicotine‐containing ECs compared with non‐nicotine ECs (also referred to as placebo).

Among the higher‐quality reviews, Levett [23] (4 studies, *n* = 1756) reported an RR of 1.56 (95% CI = 1.13–2.15, *I* ^2^ = 0%), and Lindson [10] (6 studies, *n* = 1613) found an RR of 1.46 (95% CI = 1.09–1.96, *I* ^2^ = 4%). The component network meta‐analysis (CNMA) reported by Lindson [25], including 16 studies (*n* = 3828), found nicotine ECs more effective than placebo (OR = 2.37, 95% CI = 1.73–3.24). The network meta‐analysis performed by Thomas [29], involving 363 studies (*n* = 201 045), reported an OR of 3.22 (95% CI = 1.63–6.36) for high‐dose nicotine ECs versus placebo. For low‐dose nicotine ECs, the pooled OR was 3.22 (95% CI = 0.97–12.55), with a wide 95% CI including the possibility of no difference.

Among the lower‐quality reviews, Vanderkam [30] (4 studies, *n* = 1006) reported an RR of 1.66 (95% CI = 1.01–2.73, *I* ^2^ = 0%), and Khoudigian [22] (2 studies, *n* = 662) reported an RR of 2.02 (95% CI = 0.97–4.22, *I* ^2^ = 0%), with the 95% CI encompassing the possibility of a modest benefit and little or no difference. Chan [18] (5 studies, *n* not reported) compared nicotine ECs with nicotine‐free controls (placebo ECs or non‐pharmacological support), finding an RR of 2.09 (95% CI = 1.46–2.99, *I* ^2^ = 42%), and included shorter smoking cessation outcomes, with two studies reporting outcomes at less than 6 months.

Across these reviews, the RRs ranged from 1.46 to 3.22, with all point estimates suggesting higher cessation rates among users of nicotine‐containing ECs compared with non‐nicotine ECs. The direction of effect was similar across higher‐ and lower‐quality reviews, with no clear difference by review quality.

Only two reviews reported by Lindson [10, 25] provided a formal certainty of evidence assessment, rating the evidence as being of moderate and high certainty, respectively.

#### Nicotine ECs versus behavioural or no support

Two systematic reviews evaluated the effectiveness of nicotine‐containing ECs compared with behavioural support.

Lindson [10], in a higher‐quality review (9 studies, *n* = 5024), found nicotine ECs more effective than behavioural support or no support (RR = 1.88, 95% CI = 1.56–2.25, *I* ^2^ = 0%), with low certainty of evidence. Huang [20], in a lower‐quality review (1 study, *n* = 280), reported an RR of 1.51 (95% CI = 1.03–2.21, *I* ^2^ = 0%) for nicotine ECs versus behavioural support only; serious methodological concerns were raised with this meta‐analysis owing to the double counting of participants without appropriate adjustment.

#### Nicotine ECs versus mixed interventions

Two reviews compared nicotine ECs with a range of non‐EC interventions.

Levett [23], in a higher‐quality review, analysed five studies with 2482 participants and reported an RR of 1.77 (95% CI = 1.29–2.44, *I* ^2^ = 0%) comparing ECs with controls, which included a combination of comparators (NRT, counselling and telephone support). Patnode [26] narratively synthesised five studies comparing nicotine ECs with non‐EC interventions but did not conduct a meta‐analysis; they reported that this was because of the small number of studies. They report mixed results on the effectiveness of ECs for smoking cessation when compared with placebo devices or NRT.

#### Nicotine ECs combined with NRT versus NRT or NRT combination therapies

Lindson [10], in a higher‐quality review, compared nicotine ECs combined with NRT and non‐nicotine ECs combined with NRT across two studies with 1039 participants, reporting an RR of 1.77 (95% CI = 1.07–2.94, *I* ^2^ = 0%). In the same review, nicotine ECs combined with NRT were compared with NRT alone across two studies with 980 participants (RR = 3.53, 95% CI = 1.93–6.44, *I* ^2^ = 0%).

#### Nicotine ECs versus varenicline

Lindson [10] (1 study, *n* = 54), in a higher‐quality review, reported an RR of 0.31 (95% CI = 0.11–0.82), suggesting varenicline may be more effective. However, the small sample size and the fact that the study was judged to be at high risk of bias (in part owing to inconsistencies in the data presented) limit any conclusions that can be drawn from this.

#### Non‐nicotine ECs versus NRT or mixed interventions

Three reviews examined the effectiveness of non‐nicotine ECs compared with NRT or non‐EC interventions.

Among the higher‐quality reviews, Lindson [10] compared non‐nicotine ECs with NRT across two studies with 344 participants and reported an RR of 0.99 (95% CI = 0.64–1.54, *I* ^2^ = 36%), compatible with a possible increase and no difference. Levett [23] compared non‐nicotine ECs with mixed interventions (NRT, counselling and telephone support) across four studies with 1380 participants, reporting an RR of 1.37 (95% CI = 0.66–2.85, *I* ^2^ = 0%), with estimates incorporating the possibility of no difference.

Quigley [28], in a lower‐quality review, reported an RR of 0.65 (95% CI = 0.27–1.30) for non‐nicotine ECs compared with NRT, with estimates compatible with the possibility of no difference.

Point estimates varied and 95% CIs were broad, indicating uncertainty about whether non‐nicotine ECs meaningfully affect cessation outcomes relative to NRT or other non‐EC interventions.

#### Non‐nicotine ECs versus behavioural support or no support

Lindson [10] (2 studies, *n* = 388), in a higher‐quality review, reported an RR of 1.63 (95% CI = 0.81–3.25, *I* ^2^ = 0%), with estimates compatible with a possible benefit and little or no difference between groups.

### Adverse events (AEs) and serious adverse events (SAEs)

Table 6 summarises findings from reviews that meta‐analysed AEs and SAEs, with arrows showing the direction of effect and whether the 95% CIs included no difference. Evidence was mixed, mainly reflecting few events and imprecision. Two reviews indicated possible increases in SAEs among nicotine EC users; other estimates were compatible with little or no difference.

**TABLE 6:** Effect direction plot, adverse and serious adverse events.

Comparator	Review	Analysis type	AEs	Effect size (estimate and range)	SAEs	Effect size (estimate and range)	AMSTAR quality
Nicotine ECs vs NRT	Ibrahim (2021)	PW	▽	RR = 0.90 (0.20–3.95)	△	RR = 1.60 (0.12–22.22)	Higher
	Li (2022)	PW	△	RR = 1.20 (0.97–1.48)	△	RR = 1.29 (0.73–2.28)	Higher
	Lindson (2024)	PW	△	RR = 1.03 (0.91–1.17)	△	RR = 1.20 (0.90–1.60)	Higher
	Pound (2021)	PW	◁▷	RR = 0.96 (0.76–1.20)	—	—	Higher
	Vanderkam (2022)	PW	—	—	▲	RR = 1.53 (1.02–2.30)	Lower
Nicotine ECs vs non‐nicotine ECs/placebo	Khoudigian (2016)	PW	△	MD = 0.09 (−0.28–0.46)	—	—	Lower
	Levett (2023)	PW	—	—	▽	RR = 0.78 (0.18–3.35)	Higher
	Lindson (2024)	PW	◁▷	RR = 1.01 (0.91–1.11)	◁▷	RR = 1.00 (0.56–1.79)	Higher
	Vanderkam (2022)	PW	—	—	△	RR = 1.18 (0.65–2.16)	Lower
	Lindson (2023)	CNMA	—	—	▽	OR = 0.79 (0.50–1.23)	Higher
Nicotine ECs vs behavioural or no support	Lindson (2024)	PW	▲	RR = 1.22 (1.12–1.32)	▽	RR = 0.89 (0.59–1.34)	Higher
Nicotine ECs vs mixed interventions	Levett (2023)	PW	—	—	△	RR = 1.42 (0.83–2.43)	Higher
Nicotine ECs + NRT vs non‐nicotine ECs + NRT	Lindson (2024)	PW	△	RR = 1.11 (0.93–1.32)	▽	RR = 0.66 (0.38–1.14)	Higher
Nicotine ECs + NRT vs NRT	Lindson (2024)	PW	◁▷	RR = 0.96 (0.83–1.11)	△	RR = 1.26 (0.46–3.42)	Higher
Non‐nicotine ECs vs mixed interventions	Levett (2023)	PW	—	—	▲	RR = 1.61 (1.17–2.20)	Higher

*Note*: ▲ = greater quit rates in EC arm, 95% CI does not include possibility of no difference; ▼ = fewer quit rates in EC arm, 95% CI does not include possibility of no difference; ◁▷ = no change/mixed effects/conflicting findings; △ = greater quit rates in EC arm, but 95% CI includes possibility of no difference; ▽ = fewer quit rates in EC arm, but 95% CI includes possibility of no difference; AE = adverse event; AMSTAR = A MeaSurement Tool to Assess systematic Reviews; CNMA = component network meta‐analysis; EC = electronic cigarette; NMA = network meta‐analysis; NRT = nicotine replacement therapy; PW = pairwise comparison; SAE = serious adverse event.

### Adverse events

#### Nicotine ECs versus NRT

Four systematic reviews evaluated AEs associated with nicotine‐containing ECs compared with NRT.

Among the higher‐quality reviews, Ibrahim [21] (3 studies, *n* = 1107) reported an RR of 0.90 (95% CI = 0.20–3.95), with substantial between‐study inconsistency (*I* ^2^ = 57%). Li [24] (5 studies, *n* = 1684) found an RR of 1.20 (95% CI = 0.97–1.48, *I* ^2^ = 44%), showing moderate inconsistency. Lindson [10] (5 studies, *n* = 2052) reported an RR of 1.03 (95% CI = 0.91–1.17), with no evidence of inconsistency (*I* ^2^ = 0%). Pound [27] (4 studies, *n* = 758) found an RR of 0.96 (95% CI = 0.76–1.20), with moderate inconsistency (*I* ^2^ = 42).

Quigley [28], in a lower‐quality review, did not provide quantitative data but narratively summarised AEs, noting no SAEs related to the investigated products.

Across these reviews, the RR ranged from 0.90 to 1.20, with estimates generally compatible with little or no difference in AE rates between nicotine ECs and NRT. The reported *I* ^2^ values indicated inconsistency ranging from low to substantial across reviews, and the certainty of evidence was rated from very low [24, 27] to moderate [10].

#### Nicotine ECs versus non‐nicotine ECs/placebo

Two reviews examined AEs associated with nicotine ECs compared with non‐nicotine ECs. Khoudigian [22], in a lower‐quality review (2 studies, *n* = 441), reported a mean difference of 0.09 (95% CI = −0.28, 0.46), with moderate between‐study inconsistency (*I* ^2^ = 53%). Lindson [10] (5 studies, *n* = 840), in a higher‐quality review, reported an RR of 1.01 (95% CI = 0.91–1.11, *I* ^2^ = 0%), based on evidence of moderate certainty.

#### Nicotine ECs versus behavioural or no support

Two reviews evaluated AEs associated with nicotine ECs compared with behavioural support or no support. Huang [20], in a lower‐quality review, included one study but did not provide quantitative data. Lindson [10], in a higher‐quality review (4 studies, *n* = 765), reported an RR of 1.22 (95% CI = 1.12–1.32, *I* ^2^ = 41%) and a low certainty of evidence. The pooled estimate from the review suggests a possible small increase in participants reporting AEs using nicotine ECs compared with behavioural support alone.

#### Nicotine ECs versus heated tobacco

Lindson [10], in a higher‐quality review (1 study, *n* = 220), reported an RR of 0.86 (95% CI = 0.68–1.10), with estimates compatible with little or no difference in the rates of AEs for the two products.

#### Nicotine ECs combined with NRT versus NRT or non‐nicotine ECs

Lindson [10], in a higher‐quality review, compared nicotine ECs combined with NRT and non‐nicotine ECs combined with NRT (2 studies, *n* = 677), and found an RR of 1.11 (95% CI = 0.93–1.32, *I* ^2^ = 0%). In the same review, nicotine ECs + NRT was compared with NRT (3 studies, *n* = 1984), reporting an RR of 0.96 (95% CI = 0.83–1.11, *I* ^2^ = 64%), showing substantial between‐study inconsistency. Across both comparisons, estimates were compatible with little or no difference in AE rates between groups.

### Serious adverse events

#### Nicotine ECs versus NRT

Four systematic reviews evaluated SAEs associated with nicotine‐containing ECs compared with NRT.

Among the higher‐quality reviews, Ibrahim [21] (2 studies, *n* = 1781) reported an RR of 1.60 (95% CI = 0.12–22.22, *I* ^2^ = 0%), Li (5 studies, *n* = 1684) found an RR of 1.29 (95% CI = 0.73–2.28), showing moderate between‐study inconsistency (*I* ^2^ = 41%), and Lindson [10] (6 studies, *n* = 2411) reported an RR of 1.20 (95% CI = 0.90–1.60), with moderate inconsistency across studies (*I* ^2^ = 32%).

The lower‐quality review, Vanderkam [30] (3 studies, *n* = 1618), found an RR of 1.53 (95% CI = 1.02–2.30, *I* ^2^ = 13%), suggesting a possible increase in SAEs among users of nicotine ECs, but noted that none of the SAEs reported were directly connected to the use of nicotine ECs.

The RRs ranged from 1.20 to 1.60, with most 95% CIs encompassing a possible small increase in SAEs and little or no difference between groups. Reported *I* ^2^ values indicated inconsistency ranging from none to moderate and, where assessed, the certainty of evidence was low and very low, respectively [10, 24].

#### Nicotine ECs versus non‐nicotine ECs/placebo

Five reviews examined SAEs associated with nicotine ECs compared with non‐nicotine ECs.

Among the higher‐quality reviews, Lindson [10] (9 studies, *n* = 1412) reported an RR of 1.00 (95% CI = 0.56–1.79, *I* ^2^ = 0%), with estimates compatible with little or no difference in SAEs between groups. Another higher‐quality review, Lindson [25] (7 studies, *n* = 1642), reported an OR of 0.79 (95% CI = 0.50–1.23) for SAEs at 1 week or longer when comparing nicotine ECs with placebo, and rated the certainty of the evidence as low.

Among the lower‐quality reviews, Khoudigian [22] did not conduct a meta‐analysis for SAEs as they only included one study that reported SAEs, while Vanderkam [30] (5 studies, *n* = 1447) found an RR of 1.18 (95% CI = 0.65–2.16, *I* ^2^ = 0%), with estimates compatible with a possible increase and no difference. Levett [23] (4 studies, *n* = 2367) reported an RR of 0.78 (95% CI = 0.18–3.35) when comparing nicotine ECs with non‐nicotine ECs, indicating wide uncertainty and compatibility with no difference.

#### Nicotine ECs versus mixed interventions

One lower‐quality review, Levett [23] (4 studies, *n* = 2367), compared nicotine ECs to mixed interventions (NRT, counselling and telephone support) and reported an RR of 1.42 (95% CI = 0.83–2.43), consistent with little or no difference.

#### Nicotine ECs versus behavioural or no support

In a higher‐quality review (10 studies, *n* = 3263) comparing nicotine ECs versus behavioural or no support, Lindson reported an RR of 0.89 (95% CI = 0.59–1.34), with moderate between‐study inconsistency (*I* ^2^ = 42%) and very low certainty of evidence [10].

#### Nicotine ECs versus heated tobacco or varenicline

Lindson [10], a higher‐quality review (1 study, *n* = 220), compared nicotine ECs with heated tobacco and (1 study, *n* = 54) compared nicotine ECs with varenicline. Neither comparison provided estimable RRs for SAEs.

#### Nicotine ECs combined with NRT versus NRT or non‐nicotine ECs

Lindson [10], in a higher‐quality review (2 studies, *n* = 1069), compared nicotine ECs combined with NRT against non‐nicotine ECs combined with NRT and found an RR of 0.66 (95% CI = 0.38–1.14, *I* ^2^ = 0%). In the same review, nicotine ECs combined with NRT were compared with NRT alone (4 studies, *n* = 2245), reporting an RR of 1.26 (95% CI = 0.46–3.42, *I* ^2^ = 64%), showing substantial between‐study inconsistency. Across comparisons, estimates were compatible with a possible small increase and little or no difference in SAEs between groups.

#### Nicotine ECs versus mixed interventions

Patnode [26], in a lower‐quality review, narratively stated ‘Nine trials reported on the potential short‐term harms of e‐cigarette use for cessation; none suggested relatively higher rates of serious adverse events’.

#### Non‐nicotine ECs versus mixed interventions

Levett [23], in a higher‐quality review (3 studies, *n* = 1240), compared non‐nicotine ECs with mixed interventions and reported an RR of 1.61 (95% CI = 1.17–2.20, *I* ^2^ not reported), with the 95% CI suggesting a possible increase in SAEs among users of non‐nicotine ECs.

### Other outcomes

Data on heart rate, blood pressure, carbon monoxide levels, lung function tests, oxygen saturation, toxins and carcinogens were reported only by Lindson [10]. Please refer to Lindson [10], or subsequent updates of this review, for these outcomes.

## RESULTS: EGM

We included 90 trials and 14 systematic reviews (for the PRISMA diagram, see Appendix S1). The interactive EGM is available in the supporting information (Appendix S4), with comparator type(s) and outcome categories organised in the rows and columns headings. The EGM glossary is available on our project webpage (see Appendix S3).

The main clusters of evidence were concentrated in studies comparing the effectiveness of nicotine ECs versus NRT, or nicotine ECs versus behavioural support only or no support, for smoking cessation at 6 months or longer, AEs at 1 week or longer and carbon monoxide at 1 week or longer. Table 7 lists the main comparator–outcome gaps shown in the EGM. Absolute gaps refer to comparator–outcome combinations where no studies were found, whereas relative gaps refer to those combinations where 10 or fewer studies were identified. For the full reporting of EGM results, namely distribution by characteristics of interest, please see Appendix S4.

**TABLE 7:** Comparator–outcome gaps.

Comparator–outcome absolute gaps
**Impact of the following comparators on any outcome of interest**
Nicotine ECs versus cytisine Nicotine ECs versus bupropion Nicotine ECs versus nicotine pouches Nicotine ECs + cytisine versus cytisine Nicotine ECs + bupropion versus bupropion Nicotine ECs + varenicline versus varenicline

Comparator–outcome relative gaps
**Impact of the following comparators on any outcome of interest**
Nicotine ECs versus varenicline Nicotine ECs versus heated tobacco Comparisons based on flavours Comparisons based on device type Nicotine salt versus free‐base nicotine ECs Non‐nicotine ECs versus behavioural support only/no support Non‐nicotine ECs + NRT versus NRT Non‐nicotine ECs versus NRT Advice to use ECs compared with no advice to use e‐cigarettes Nicotine ECs + NRT versus non‐nicotine ECs + NRT Nicotine ECs + NRT versus NRT Nicotine ECs versus nicotine‐free control High‐dose nicotine ECs versus control Low‐dose nicotine ECs versus control
I**mpact of any of the comparators on the following outcomes**
Heart rate at ≥1 week Systolic blood pressure at ≥1 week Blood oxygen saturation at ≥1 week Lung function (e.g. FEV) at ≥1 week Toxicants (e.g. 3‐HPMA,2‐HPMA, AAMA, HMPMA, NNAL) at ≥1 week

Abbreviations: EC = electronic cigarette; FEV = forced expiratory volume; NRT = nicotine replacement therapy.

## DISCUSSION

### Summary of key findings

To the best of our knowledge, this is the first overview of reviews and EGM of ECs for smoking cessation. We included 14 systematic reviews, with seven of higher quality and with seven of lower quality. Findings from higher‐quality reviews were consistent, indicating greater cessation with nicotine‐containing ECs than with other interventions, whereas lower‐quality reviews produced more variable and imprecise estimates. When restricted to higher‐quality evidence, the results consistently favoured nicotine ECs over NRT, non‐nicotine ECs and other comparators. Twenty‐one meta‐analyses all reported point estimates favouring nicotine ECs. Direct evidence comparing nicotine ECs with varenicline is extremely limited, with only a single small trial at high risk of bias identified, while indirect evidence from a CNMA [25] does not indicate a clear difference in quit rates between the two interventions, with both associated with higher cessation rates than placebo.

For AEs, pooled estimates generally suggested little or no difference between groups, with 95% CIs compatible with equivalence and small differences. One higher‐quality review [10] reported increased AEs with nicotine ECs versus behavioural support alone, but with evidence of low certainty. For SAEs, four reviews reported point estimates indicating possible increases; however, all but one had wide 95% CIs spanning the possibility of no difference or small increases. The only review presenting an interval above the null [30] was a lower‐quality review that included three studies, with all also included in [10], and used number‐randomised rather than complete‐case denominators. With very few SAEs, the two trials contributing estimable data reported on by Vanderkam [30] both showed more events in the EC arm, whereas the wider set reported by Lindson [10] showed mixed directions of effect. These differences in study inclusion, denominators and chance distribution of rare events plausibly explain the variability across reviews. Apparent differences likely reflect statistical imprecision and methodological variation rather than substantive inconsistency in the underlying evidence.

In our EGM, we mapped 90 primary studies and identified absolute gaps in evidence comparing the effects of nicotine ECs with cytisine, bupropion and nicotine pouches. Most available studies used data from high‐income countries.

### Strengths and limitations

This overview followed rigorous methods and best‐practice guidance. It forms part of the programme supporting the Cochrane LSR of ECs for smoking cessation [10]. Although we pre‐registered our plans and adhered to best practice to minimise bias towards the Cochrane review, some influence cannot be ruled out. Our work is consistent with inclusion criteria for the Cochrane review; however, another research team might have used different criteria (e.g. including observational data) or adopted different nomenclature for the EGM and glossary. This extends to the pragmatic threshold for classifying evidence density that we adopted in our EGM. Our comparisons did not distinguish between single‐form and combination NRT, and individual reviews varied in how they defined individual studies. Our searches were run in April 2024, meaning subsequent studies and reviews may have been missed. We do not think this would affect the consistency of the observed cessation outcomes across meta‐analyses, but it does mean that relevant primary studies may have been missed. This is a fast‐moving field in which new research is regularly published, meaning that there will inevitably be a lag between literature reviews and the most recent publications. Our EGM does not include ongoing studies as that is not standard practice and would require a different data structure, and we encourage readers to look at the latest version of the Cochrane LSR (which is updated regularly and includes links to new primary and ongoing studies) for the most up‐to‐date picture of evidence on this topic. Monthly search findings are available on the website.

## CONCLUSION

This overview extends the Cochrane living review by synthesising evidence across multiple systematic reviews, highlighting consistency and divergence in findings. Our hope is that this overview and EGM can lay to rest some claims that evidence is ‘mixed’ regarding the impacts of ECs on smoking abstinence, given the clear consistency of results across meta‐analyses. We also hope they can be useful tools to inform research in this space. As evidenced from the meta‐analyses of SAE data, evidence on SAEs remains largely inconclusive and mixed, in part owing to the fact this is a rare outcome and hence most studies are underpowered to detect effects. Future primary research should continue to collect and report data on SAEs, as well as expand its geographical distribution to low‐and middle‐income countries.

## AUTHOR CONTRIBUTIONS

**Angela Difeng Wu:** Conceptualization (equal); data curation (lead); methodology (equal); visualization (lead); writing—original draft (lead); writing—review and editing (equal). **Monserrat Conde:** Data curation (equal); visualization (equal); writing—original draft (lead); writing—review and editing (equal). **Ailsa R. Butler:** Conceptualization (equal); data curation (supporting); funding acquisition (supporting); methodology (equal); writing—review and editing (equal). **Ethan Knight:** Data curation (supporting); writing—review and editing (equal). **Nicola Lindson:** Conceptualization (equal); data curation (supporting); funding acquisition (lead); methodology (equal); writing—review and editing (equal). **Jonathan Livingstone‐Banks:** Conceptualization (equal); data curation (supporting); methodology (equal); writing—review and editing (equal). **Peter Hajek:** Conceptualization (supporting); methodology (supporting); writing—review and editing (equal). **Hayden McRobbie:** Conceptualization (supporting); methodology (supporting); writing—review and editing (equal). **Rachna Begh:** Conceptualization (supporting); methodology (supporting); writing—review and editing (equal). **Annika Theodoulou:** Conceptualization (supporting); methodology (supporting); writing—review and editing (equal). **Caitlin Notley:** Conceptualization (supporting); methodology (supporting); writing—review and editing (equal). **Tari Turner:** Conceptualization (supporting); methodology (supporting); writing—review and editing (equal). **Eliza Zhitnik:** Data curation (supporting); writing—review and editing (equal). **Jamie Hartmann‐Boyce:** Conceptualization (equal); data curation (supporting); funding acquisition (lead); methodology (equal); writing—review and editing (equal).

## DECLARATION OF INTERESTS

A.D.W., none known; M.C., none known; J.H.B. is paid for research consultancy from the Truth Initiative; N.L. is an associate editor for *Addiction*; J.L.B., none known; P.H., none known; A.R.B., none known; C.N. has received an honorarium from Vox Media for filming a ‘nicotine explainer’ on the role of nicotine in addiction; A.T., none known; R.B., none known; T.T., none known; H.M., none known; E.K., none known; E.Z., none known.

## Supporting information


**Appendix S1.** Search strategy, EGM coding categories and PRISMA flow chart.

**Appendix S2.**Template data extraction form.

**Appendix S3.** EGM glossary.

**Appendix S4.** EGM ECSC.

**Appendix S5.** Descriptive results of EGM.

## Data Availability

The data that support the findings of this study are available from the corresponding author upon reasonable request.
