# Supplementary material for: Electronic cigarettes for smoking cessation: An overview of systematic reviews and evidence and gap map
Source: Addiction. 2026 Mar 26;121(8):1957–71. doi: 10.1111/add.70388 (PMC13357930; doi:10.1111/add.70388)
Supplement: Supplementary file 1 — Appendix S1. Search strategy, EGM coding categories and PRISMA flow chart. [file ADD-121-1957-s003.docx]

Supplemental Materials 1

1. Search strategies

**1.1 Overview and EGM (systematic reviews)**

**Ovid databases (MEDLINE, Embase, PsycINFO)**

1. Meta-Analysis as Topic/

2. (meta analy$ or metaanaly$ or (systematic adj (review$1 or overview$1))).tw.

4. Meta-Analysis/

6. exp Review Literature as Topic/

5. 1 or 2 or 3 or 4

6. (Cochrane or embase or psychlit or psyclit or psychinfo or psycinfo or cinahl or cinhal or science citation index or bids or cancerlit).ab.

7. (reference list$ or bibliograph$ or hand-search$ or relevant journals or manual search$).ab.

8. (selection criteria or data extraction).ab.

9. Review/

10. 8 and 9

11. Comment/ or Letter/ or Editorial/

12. animal/

13. human/

14. 12 not (12 and 13)

15. 11 or 14

16. 5 or 6 or 7 or 10

17. 16 not 15

18. randomized controlled trial.pt. OR controlled clinical trial.pt. OR randomized.ab. OR placebo.ab. OR clinical trials as topic.sh. OR randomly.ab. OR trial.ti.

19. exp animals/ not human/

20. 18 not 19

21. Exp case control studies/ OR Exp cohort studies/ OR Case control.tw. OR (cohort adj (study or studies)).tw. OR Cohort analy$.tw. OR (Follow up adj (study or studies)).tw. OR (observational adj (study or studies)).tw. OR Longitudinal.tw.

22. e-cig*.mp. OR ecig*.mp. OR electr* cigar*.mp. OR electronic nicotine.mp. OR (vape or vapes or vaporizer or vapourizer or vaporiser or vapouriser or vaper or vapers or vaping).ti,ab. OR Exp Electronic Nicotine Delivery Systems/

23. 17 AND 22

24. 20 AND 22

25. 21 AND 22

26. 23 OR 24 OR 25

**Cochrane Database of Systematic Reviews**

#1 (e-cig* OR ecig* OR electr* cigar* OR electronic nicotine or vape or vapes or vaporizer or vapourizer or vaporiser or vapouriser or vaper or vapers or vaping):ti,ab

#2 MeSH descriptor: [Electronic Nicotine Delivery Systems] explode all trees

#3 #1 OR #2 [in Cochrane Reviews and Cochrane Protocols]

**CENTRAL**

1. (e-cig* or ecig* or electr* cigar* or electronic nicotine):ti,ab,KY,MH,EMT,KW,XKY,EH,KY

2. (vape or vapes or vaporizer or vapourizer or vaporiser or vapouriser or vaper or vapers or vaping):ti,ab,KY,MH,EMT,KW,XKY,EH,KY

3. MESH DESCRIPTOR Electronic Nicotine Delivery Systems EXPLODE ALL

4. #3 OR #2 OR #1

**1.2 EGM (primary studies, same search strategies as linked Cochrane review)**

**2020 update onwards**

**Ovid databases (MEDLINE, Embase, PsycINFO)**

1. exp case control studies/ or exp cohort studies/ or Case control.tw. or (cohort adj (study or studies)).tw. or Cohort analy$.tw. or (Follow up adj (study or studies)).tw. or (observational adj (study or studies)).tw. or Longitudinal.tw.

2. (e‐cig$ or ecig$ or electr$ cigar$ or electronic nicotine).mp. or (vape or vapes or vaporizer or vapourizer or vaporiser or vapouriser or vaper or vapers or vaping).ti,ab. or exp Electronic Nicotine Delivery Systems/

3. (randomized controlled trial or controlled clinical trial).pt. or randomized.ab. or placebo.ab. or clinical trials as topic.sh. or randomly.ab. or trial.ti.

4. exp animals/ not human/

5. 3 not 4

6. 2 and 5

7. 1 and 2

8. 6 or 7

9. smoking cessation.mp. or exp Smoking Cessation/

10. tobacco cessation.mp. or "Tobacco‐Use‐Cessation"/

11. (nicotine dependence or tobacco dependence).mp.

12. exp Smoking/th

13. "Tobacco‐Use‐Disorder"/

14. Smoking reduction/ or Smoking reduction.mp.

15. exp Pipe smoking/ or exp Tobacco smoking/ or exp Tobacco Products/

16. ((quit$ or stop$ or ceas$ or giv$ or abstain* or abstinen*) adj5 (smoking or smoke* or tobacco)).ti,ab.

17. exp Tobacco/ or exp Nicotine/

18. 9 or 10 or 11 or 12 or 13 or 14 or 15 or 16 or 17

19. 8 and 18

**CENTRAL (via CRS‐Web), (also used to search CTAG Specialised Register until March 2023)**

1. (e‐cig* or ecig* or electr* cigar* or electronic nicotine):ti,ab,KY,MH,EMT,KW,XKY,EH,KY

2. (vape or vapes or vaporizer or vapourizer or vaporiser or vapouriser or vaper or vapers or vaping):ti,ab,KY,MH,EMT,KW,XKY,EH,KY

3. MESH DESCRIPTOR Electronic Nicotine Delivery Systems EXPLODE ALL

4. #3 OR #2 OR #1

**MEDLINE search strategy ‐ pre‐2020**

e‐cig$.mp. [mp=title, abstract, original title, name of substance word, subject heading word, protocol supplementary concept, rare disease supplementary concept, unique identifier]

electr$ cigar$.mp.

electronic nicotine.mp.

(vape or vaper or vapers or vaping).ti,ab.

1 OR 2 OR 3 OR 4

Identical terms used for other databases.

Line 4 added to search strategy for 2016 update.

1. Table 1 – List of the EGM coding categories

| Table 1- List of coding categories | |
| --- | --- |
| Comparator type(s) | |
| Nicotine EC versus NRT | Nicotine containing e-cigarette compared to Nicotine Replacement Therapy |
| Nicotine EC versus varenicline | Nicotine containing e-cigarette compared to varenicline |
| Nicotine EC versus cystine | Nicotine containing e-cigarette compared to cystine |
| Nicotine EC versus bupropion | Nicotine containing e-cigarette compared to bupropion |
| Nicotine EC versus heated tobacco | Nicotine containing e-cigarette compared to heated tobacco |
| Nicotine EC versus nicotine pouches | Nicotine containing e-cigarette compared to nicotine pouches |
| Nicotine EC versus non-nicotine EC | Nicotine containing e-cigarette compared to non-nicotine e-cigarettes |
| Nicotine EC versus behavioural support only/no support | Nicotine containing e-cigarette compared to behavioural support only or no support |
| Higher versus lower nicotine content EC | Higer nicotine content e-cigarettes compared to lower nicotine content e-cigarettes |
| Comparison based on flavour | Comparisons based on the flavour of the e-liquid |
| Comparisons based on device type | Comparisons based on device type (e-cigarettes) |
| Nicotine salt EC versus free-based nicotine EC | Nicotine salt containing e-cigarette compared to free-based nicotine containing e-cigarette |
| Non-nicotine EC versus behavioural support only/no support | Non-nicotine containing e-cigarette compared behavioural support only or no support |
| Non-nicotine EC + NRT versus NRT | Non-nicotine containing e-cigarette combined with nicotine replacement therapy compared to nicotine replacement therapy |
| Advice to use e-cigarettes compared to no advice to use e-cigarettes | Advice to use e-cigarettes compared to no advice to use e-cigarettes |
| Nicotine EC + NRT versus non-nicotine + NRT | Nicotine containing e-cigarette combined with nicotine replacement therapy compared to non-nicotine e-cigarette combined with nicotine replacement therapy |
| Nicotine EC + NRT versus NRT | Nicotine containing e-cigarette combined with nicotine replacement therapy compared to nicotine replacement therapy |
| Nicotine EC + varenicline vs. varenicline | Nicotine containing e-cigarette combined with varenicline compared to varenicline |
| Nicotine EC + cytisine vs. cytisine | Nicotine containing e-cigarette combined with cystisine compared to cystisine |
| Nicotine EC + bupropion vs bupropion | Nicotine containing e-cigarette combined with bupropion compared to buproprion |
| Outcomes | |
| Smoking cessation at six months or longer | Smoking cessation at six months or longer |
| Adverse events at 1 week or longer | Adverse events at 1 week or longer |
| Serious adverse events at 1 week or longer | Serious adverse events at 1 week or longer |
| Carbon monoxide (ppm) at 1 week or longer | Carbon-monoxide at 1 week or longer |
| Heart rate (bpm) at 1 week or longer | Heart rate at 1 week or longer |
| Systolic blood at 1 week or longer | Systolic blood pressure at 1 week or longer |
| Blood oxygen saturation at 1 week or longer | Blood oxygen saturation at 1 week or longer |
| Lung function (e.g. FEV) at 1 week or longer | Lung function at 1 week or longer |
| Toxicants (e.g. 3-HPMA, 2-HPMA, AAMA, HMPM | Toxicants (e.g., cancer-related) at 1 week or longer |
| Filters | Dimensions |
| Study design | RCT  Non-randomized  Higher Quality Systematic Review  Lower Quality Systematic Review |
| Device type | Cig-a –like  Cartridge  Refillable  Pod  Disposable  Unclear |
| Sub-population | Pregnancy  Substance use disorder  Based on a mental health condition  Based on a physical health condition  Minoritized racial/ethnic group  Homelessness  Veterans  Young people (29 years or younger)  Older adults (55 years or older) |
| Country | Country(ies) where the study took place |
| WHO region | African Region (AFRO)  Region of the Americas (AMRO)  Eastern Mediterranean Region (EMRO)  European Region (EURO)  South-East Asia region (SEARO)  Western Pacific Region (WPRO)  Multiple regions  Unspecified |
| World Bank Country classification by income level (2024) | Low-income economies  Lower-middle income economies  Upper-middle income economies  Multiple economies  Unspecified |

1. PRISMA Flowcharts

Figure 1- PRISMA flowchart of selection of systematic reviews to be included in overview and EGM


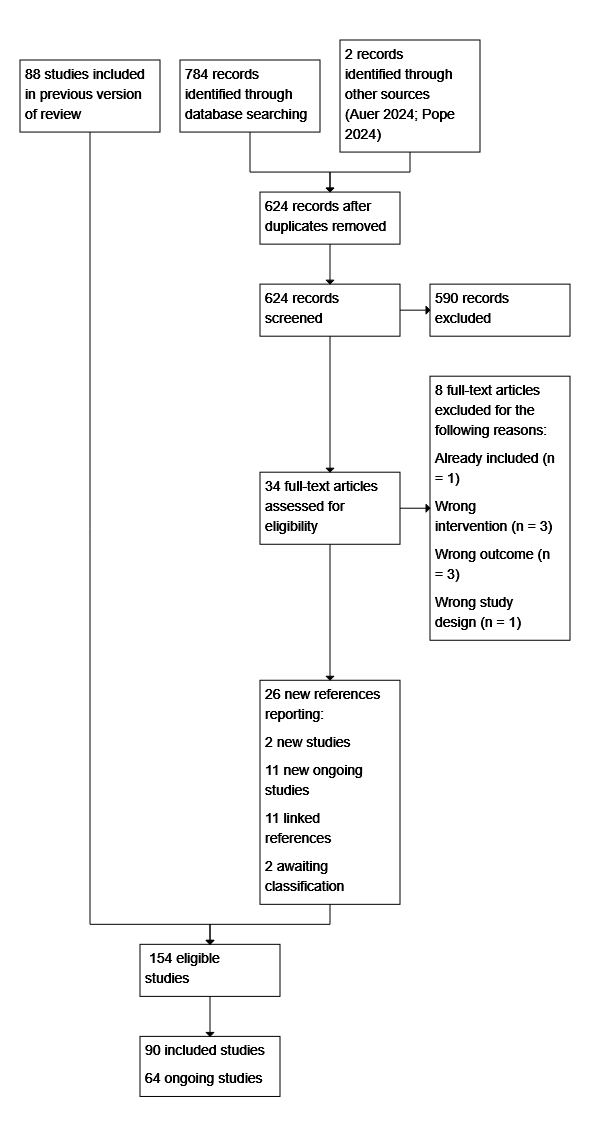


Figure 2- PRISMA flowchart of selection of primary studies to be included in EGM (same as linked Cochrane review)
