# Supplementary material for: Electronic cigarettes for smoking cessation: An overview of systematic reviews and evidence and gap map
Source: Addiction. 2026 Mar 26;121(8):1957–71. doi: 10.1111/add.70388 (PMC13357930; doi:10.1111/add.70388)
Supplement: Supplementary file 3 — Appendix S3. EGM glossary. [file ADD-121-1957-s004.docx]

# Supplementary materials 2 - Glossary

This glossary describes the definitions of the key terms of our EGM. It includes a descriptive list of overall terminology, interventions, outcome categories and filters

| **Overall terminology** | |
| --- | --- |
| **Term** | **Definition** |
| **Evidence and gap map** | “Evidence and gap maps are interactive visual presentations of available and non-existent research evidence for a given topic or policy domain. They follow rigorous methods and usually map evidence produced by systematic reviews and primary studies.”  Source: Evidence and gap maps - The Campbell Collaboration  [Electronic cigarettes and subsequent use of cigarettes in young people: An evidence and gap map - Conde - 2024 - Addiction - Wiley Online Library](https://onlinelibrary.wiley.com/doi/10.1111/add.16583?af=R) |
| **Systematic review** | Systematic reviews are studies that aim to synthetize all available evidence that meets pre-specified requirements, to answer a specific research question. The systematic methods are defined in advance.  Source:  https://systematicreviewsjournal.biomedcentral.com/articles/10.1186/2046-4053-4-1  <https://training.cochrane.org/handbook/current/chapter-i>  [Electronic cigarettes and subsequent use of cigarettes in young people: An evidence and gap map - Conde - 2024 - Addiction - Wiley Online Library](https://onlinelibrary.wiley.com/doi/10.1111/add.16583?af=R) |
| **E-cigarette(s)** | " An electronic vaping device that is handheld and produces for inhalation an aerosol formed by heating an e-liquid using a battery-powered heating coil."  Source: E-cigarette - Definition (v1) by Addiction Ontology \| Qeios |

| **Comparators** | **Definition** |
| --- | --- |
| **Nicotine EC versus NRT** | Nicotine e-cigarettes compared to Nicotine Replacement Therapy  Nicotine replacement therapy is a therapeutic use of nicotine containing medications. (E.g, gum, transdermal patch, nasal spray, oral inhaler, tablets). |
| **Nicotine EC versus varenicline** | Nicotine e-cigarettes compared to varenicline |
| **Nicotine EC versus cytisine** | Nicotine e-cigarettes compared to cytisine |
| **Nicotine EC versus bupropion** | Nicotine e-cigarettes compared to bupropion |
| **Nicotine EC versus heated tobacco** | Nicotine e-cigarettes compared to heated tobacco |
| **Nicotine EC versus nicotine pouches** | Nicotine e-cigarettes compared to nicotine pouches |
| **Nicotine EC versus non-nicotine EC** | Nicotine e-cigarettes compared to non-nicotine e-cigarettes |
| **Nicotine EC versus behavioural support only/no support** | Nicotine e-cigarettes compared to behavioural support only or no support |
| **Higher versus lower nicotine content ECs** | Higher compared to lower nicotine content electronic cigarettes |
| **Comparisons based on flavour** | N/A |
| **Comparisons based on device type** | N/A |
| **Nicotine salts EC versus free-base nicotine EC** | Nicotine salts e-cigarettes compared to free-base nicotine e-cigarettes |
| **Non-nicotine EC versus behavioural support only/no support** | Non-nicotine e-cigarettes compared to behavioural support only or no support |
| **Non-nicotine EC + NRT versus NRT** | Non-nicotine e-cigarettes combined with Nicotine Replacement Therapy compared to Nicotine Replacement Therapy |
| **Non-nicotine EC versus NRT** | Non-nicotine e-cigarettes compared to Nicotine Replacement Therapy |
| **Advice to use e-cigarettes compared to no advice to use e-cigarettes** | N/A |
| **Nicotine EC + NRT versus non-nicotine EC + NRT** | Nicotine e-cigarettes combined with Nicotine Replacement Therapy compared to non-nicotine e-cigarettes with Nicotine Replacement Therapy |
| **Nicotine EC + NRT versus NRT** | Nicotine e-cigarettes combined with Nicotine Replacement Therapy compared to Nicotine Replacement Therapy |
| **Nicotine EC + varenicline vs varenicline** | Nicotine e-cigarettes combined with varenicline compared to varenicline |
| **Nicotine EC + cytisine vs. cytisine** | Nicotine e-cigarettes combined with cytisine compared to cytisine |
| **Nicotine EC + bupropion vs bupropion** | Nicotine e-cigarettes combined with bupropion compared to bupropion |
| **Nicotine EC versus Nicotine Free control** | Nicotine e-cigarettes compared to Nicotine free control |
| **High Dose Nicotine EC versus Control** | High Dose Nicotine e-cigarettes compared to control |
| **Low Dose Nicotine EC versus Control** | Low Dose Nicotine e-cigarettes compared to control |

| **Outcomes** | **Definition** |
| --- | --- |
| **Vaping cessation at 6 months or longer** | Vaping cessation at least six months from the start of the intervention.  Source: https://doi.org/10.1002/14651858.CD010216.pub9 |
| **Adverse events at 1 week or longer** | Number of participants reporting adverse events (AEs) at one week or longer (as defined by study authors).  Source: https://doi.org/10.1002/14651858.CD010216.pub9 |
| **Serious adverse events at 1 week or longer** | Number of participants reporting serious adverse events (SAEs) at one week or longer (as defined by the study authors).  Source: https://doi.org/10.1002/14651858.CD010216.pub9 |
| **Carbon monoxide (ppm) at 1 week or longer** | Changes in Carbon monoxide, as measured through blood or breath, at one week or longer  Source: https://doi.org/10.1002/14651858.CD010216.pub9 |
| **Heart rate (bpm) at 1 week or longer** | Changes to heart rate (bpm) at one week of longer  Source: https://doi.org/10.1002/14651858.CD010216.pub9 |
| **Systolic blood pressure at 1 week or longer** | Changes to systolic blood pressure (mm/hg) at one week of longer  Source: https://doi.org/10.1002/14651858.CD010216.pub9 |
| **Blood oxygen saturation at 1 week or longer** | Changes in blood oxygen saturation at one week of longer  Source: https://doi.org/10.1002/14651858.CD010216.pub9 |
| **Lung function (e.g. FEV) at 1 week or longer** | Changes in lung function measures (E.g., FEV1) at one week or longer  Source: https://doi.org/10.1002/14651858.CD010216.pub9 |
| **Toxicants (e.g. 3-HPMA,2-HPMA, AAMA, HMPMA, NNAL) at 1 week or longer** | Toxins/carcinogens as measured through blood or urine at one week or longer.  Source: https://doi.org/10.1002/14651858.CD010216.pub9 |
| **Study product use at 6 months or longer at 1 week or longer** | Changes in alcohol use status at one week or longer  Source: https://doi.org/10.1002/14651858.CD010216.pub9 |

| **Main filter** | **Sub-categories** | **Definition** |
| --- | --- | --- |
| **Funding** | **Not reported.** | N/A. |
|  | **No tobacco/vaping industry funding** |  |
|  | **Tobacco/vaping industry funding** |  |
| **Study design** | **Randomized Controlled Trial (RCT)** | Prospective, comparative study performed under controlled conditions with random allocation of interventions to the groups being compared to minimize bias and establish cause-effect relation between an intervention and an outcome.  Source: https://www.sciencedirect.com/science/article/pii/B9780323884235000960  We also map pilot trials/RCTS and feasibility RCTS/studies under this category. |
|  | **Non-randomized** | Study designs that do not include randomization of participants into the comparator groups. |
|  | **Higher quality systematic review** | Systematic review rated as higher quality based on whether they were rated “yes” or partial “yes” on six of the seven critical domains of AMSTAR-2 |
|  | **Lower quality systematic review** | Systematic review rated as higher quality based on whether they were rated “yes” or partial “yes” on six of the seven critical domains of AMSTAR-2. |
|  | **Dual users** | Participants vape and smoke at baseline. |
| **Device type** | **Cig-a-like** | An electronic cigarette that mimics the look and feel of a combustible tobacco cigarette. |
|  | **Cartridge** | A replaceable or refillable component that attaches to a separate battery. |
|  | **Refillable** | A vape in which the e-liquid can be refilled. |
|  | **Pod** | A type of vape that has a pod instead of a vape tank. |
|  | **Disposable** | A single use device that comes pre-filled with e-liquid and has a built-in battery. |
|  | **Unclear** | N/A |
|  | **Multiple** | Two or more device types. |
| **Flavours** | **Tobacco** | N/A |
|  | **Mint/menthol** | N/A |
|  | **Participants offered choice** | N/A |
|  | **Sweet (including fruit)** | N/A |
|  | **Tobacco only** | N/A |
|  | **Multiple** | Two or more flavors. |
|  | **Not reported** | N/A |
| **Sub-populations** | **Pregnancy** | N/A |
|  | **Substance use** | N/A |
|  | **Homelessness** | N/A |
|  | **Based on physical health condition** | N/A |
|  | **Based on mental health condition** | N/A |
|  | **Minoritized racial/ethnic group** | N/A |
|  | **Young people (19 years or younger)** | N/A |
|  | **Older adults (55 years or older)** | N/A |
|  | **Veterans** | Military veterans |
| **Country** | **Country where the data of the study derives from** | N/A |
|  | **Multiple** | The study uses data from two or more countries. |
|  | **Unclear** | N/A |
