# Supplementary material for: Electronic cigarettes for smoking cessation: An overview of systematic reviews and evidence and gap map
Source: Addiction. 2026 Mar 26;121(8):1957–71. doi: 10.1111/add.70388 (PMC13357930; doi:10.1111/add.70388)
Supplement: Supplementary file 5 — Appendix S5. Descriptive results of EGM. [file ADD-121-1957-s002.docx]

# Supplementary materials 3

**Descriptive results of the evidence and gap map (EGM)**

Here we summarize the main results of the evidence and gap that are not described in the main manuscript.

We included 90 primary studies in this EGM, but Kumral 2016* did not report any relevant outcomes. Therefore, the EPPI-mapper software does not display it.

**Sub-groups of the population**

All included studies focussed on adults, with one focusing on older adults. Two studies explored smoking cessation in pregnancy. Four studies in people dealing with substance use, seven in people with a physical condition, and six in people with a mental condition. Three studies looked into veterans, whereas only two studies looked into minoritized groups of the population.

**Flavours**

Ninety-five studies reported the flavours offered to the participants. Tobacco was the most frequent flavour (n=35) followed by participant offered choice, and mint/menthol flavour (n=22).

**Geographic and economic distribution**

Forty-one studies took place in the USA, followed by 18 studies in the UK, 12 using data from multiple countries and 10 being conducted in Italy. Forty-six included studies did not report the country where the data was originated from. The rest of the studies has a wide geographical distribution. Kumral 2016 was based in Turkey, being the only study with data originated from an upper-middle-income country. All other studies used data originated from high-income countries.

* Reference: Kumral TL, Saltürk Z, Yildirim G, Uyar Y, Berkiten G, Atar Y, et al. How does electronic cigarette smoking affect sinonasal symptoms and nasal mucociliary clearance? B-ENT 2016;12(1):17-21.
